# The effect of internet-administered support (carer eSupport) on preparedness for caregiving in informal caregivers of patients with head and neck cancer compared with support as usual: a study protocol for a randomized controlled trial

**DOI:** 10.1186/s12885-024-12273-y

**Published:** 2024-04-18

**Authors:** Birgitta Johansson, Åsa Cajander, Awais Ahmad, Emma Ohlsson-Nevo, Per Fransson, Brith Granström, Louise von Essen, Ulrica Langegård, Mona Pettersson, Anna Henriksson, Ylva Tiblom Ehrsson

**Affiliations:** 1 Department of Immunology, Genetics, and Pathology, Uppsala University, Rudbecklaboratoriet, 75185 Uppsala, Sweden; 2 Division of Visual Information and Interaction, Department of Information Technology, Uppsala University, 751 05 Uppsala, Box 337, Sweden; 3 Department of Surgery, Faculty of Medicine and Health, Örebro University, 701 82 Örebro, Sweden; 4 University Health Care Research Center, Faculty of Medicine and Health, Örebro University, 701 82 Örebro, Sweden; 5 Department of Nursing, Umeå University, 901 87 Umeå, Sweden; 6 Department of Diagnostics and Intervention, Umeå University, 901 87 Umeå, Sweden; 7 Healthcare Sciences and e-Health, Department of Women’s and Children’s Health, Uppsala University, Dag Hammarskjölds väg 14B, 751 85 Uppsala, Sweden; 8 Department of Oncology, Sahlgrenska University Hospital, 413 45 Gothenburg, Sweden; 9 Department of Public Health and Caring Sciences, Uppsala University, 751 22 Uppsala, Box 564, Sweden; 10 Physiotherapy and behavioral medicine, Department of Women’s and Children’s Health, Uppsala University, 751 22 Uppsala, Box 564, Sweden; 11 The School of Health, Care and Social Welfare, Mälardalen University, 721 23 Västerås, Box 883, Sweden; 12 Department of Surgical Sciences, Section of Otorhinolaryngology and Head & Neck Surgery, Uppsala University, Akademiska sjukhuset, ingång 70, bv, Rudbecklaboratoriet, 75185 Uppsala, Sweden

**Keywords:** Head and neck cancer, Informal caregivers, Internet-administered support, Preparedness for caregiving, Caregiver burden, Quality of life.

## Abstract

**Background:**

Informal caregivers (ICs) of patients with cancer provide essential and mainly uncompensated care. A self-perceived preparedness to care for the patient is associated with a lower caregiver burden, described as the extent to which caregiving is perceived as having adverse effects on IC functioning and well-being. ICs’ well-being is associated with patient-perceived quality of care, suggesting that interventions to optimize ICs’ health are essential in order to improve patient care. Head and neck cancer (HNC) is the seventh most common malignant disease in the world. The disease and its treatment have a significant negative impact on the patient’s health and quality of life. Symptoms usually interfere with swallowing, food and fluid intake, breathing, speaking, and communication. ICs frequently manage patients’ symptoms and side effects, especially problems related to nutrition and oral pain, without being properly prepared. Carer eSupport is an Internet-administered intervention, based on focus group discussions with ICs, developed in collaboration with ICs and healthcare professionals, tested for feasibility, and deemed feasible. This study protocol outlines the methods of investigating the effects of Carer eSupport plus support as usual (SAU) on self-reported preparedness for caregiving, caregiver burden, and well-being in the ICs of patients with HNC, compared with ICs receiving SAU only.

**Methods and analysis:**

In this randomized controlled trial, 110 ICs of patients with HNC, undergoing radiotherapy combined with surgery and/or medical oncological treatment, will be randomized (1:1) to Carer eSupport plus SAU or SAU only. Data will be collected at baseline (before randomization), post-intervention (after 18 weeks), and 3 months after post-intervention. The primary outcome is self-reported preparedness for caregiving. Secondary outcomes are self-reported caregiver burden, anxiety, depression, and health-related quality of life. The effect of Carer eSupport plus SAU on preparedness for caregiving and secondary outcomes, compared with SAU only, will be evaluated by intention to treat analyses using linear regression models, mixed-model regression, or analysis of covariance.

**Discussion:**

If proven effective, Carer eSupport has the potential to significantly improve ICs’ preparedness for caregiving and their wellbeing, thereby improving patient-perceived quality of care and patient wellbeing.

**Trial registration:**

ClinicalTrials.gov; NCT06307418, registered 12.03.2024 (https://clinicaltrials.gov/search? term=NCT06307418).

## Background

Informal caregivers (ICs) are still an unnoticed workforce in the care of patients with cancer. They are commonly family members, relatives, or close friends who provide essential and mainly uncompensated care. ICs are often thrust unprepared into the caring role, a role that is often physically, emotionally, and socially demanding and entails significant time and energy [1, 2]. Self-perceived readiness and capacity, reflecting the perceived preparedness to care for the patient, is associated with a lower caregiver burden [3]. Lack of preparedness often includes lack of knowledge and skills, concerns about one’s capacity for caring, distress, and feelings of abandonment by healthcare [4]. Caregiver burden may be described as the extent to which caregiving is perceived as having adverse effects on the caregiver’s emotional, social, financial, physical, and spiritual functioning [5]. ICs’ well-being is associated with patient-perceived quality of care, suggesting that interventions to optimize ICs’ health are essential in order to improve patient care [6].

Head and neck cancer (HNC) is the seventh most common malignant disease in the world and comprises a diverse group of cancer tumors in the upper aerodigestive tract [7]. The median age at diagnosis is approximately 60 years. However, incidence is increasing in younger persons diagnosed due to more cases of oropharyngeal squamous cell carcinoma associated with human papillomavirus (HPV). The prognosis of HNC varies considerably depending on the site of involvement and stage at diagnosis, among many other variables. The 5-year overall survival rate for patients with HNC is 70–90% in patients with stage I–II tumors and < 50% in patients with stage III–IV tumors [8]. Patients with HPV-associated HNC tend to have a better prognosis than do patients with non-HPV-associated HNC. The treatment of HNC differs according to the site of involvement, stage of disease, and comorbidity, but commonly includes single- or combined-modality treatment with external beam radiotherapy, surgery and/or chemotherapy, or targeted therapy [8]. The disease and treatment have a significant negative impact on the patient’s physical, social, and emotional health and overall quality of life. Symptoms usually interfere with vital functions such as swallowing, food and fluid intake, breathing, speaking, and communication, as well as altering facial appearance [9, 10]. ICs frequently manage patients’ symptoms and side effects, especially problems related to nutrition and oral pain, including tube feeding and pain management. Responsibility for navigating the healthcare system and arranging healthcare appointments are other tasks that often fall to the IC’s lot [11, 12]. Many ICs of patients with HNC prioritize the needs of the patient and set aside their own self-care care needs, which may lead to deteriorated IC health [1, 13]. Thus, both ICs’ and patients’ lives, as well as their relationships, may be severely affected by the cancer diagnosis.

Systematic reviews suggest that interventions for ICs of patients with cancer, including Internet-administered interventions, may increase self-efficacy and preparedness for caregiving, decrease caregiver burden, and/or improve well-being in ICs [14–18]. Studies of online interventions are still sparse [14] and limited in quality and generalizability; we have identified only one Internet-administered intervention (a pilot study, *n* = 38) addressing the ICs of patients with HNC [19]. Ugalde et al. [20] explored the potential of 26 IC interventions to be implemented in practice. Only two of the included studies involved ICs in developing the intervention, only two addressed whether the intervention was appropriate for clinical care, and no study discussed intentions or actions to facilitate implementation in routine cancer care. Thus, existing interventions lack the necessary components to bridge the gap between research and clinical care. We want to reduce the gap between scientific evidence and practical application, ultimately enhancing health outcomes for both ICs and patients by exploring the potential of Internet-administered interventions designed with ICs’ input and emphasizing the importance of application usability to cater to a diverse range of technological proficiencies and maintain long-term engagement [21, 22].

### The Carer eSupport project: development of a usable internet-administered intervention

Our multidisciplinary research group includes expertise in HNC clinical care, the caring sciences, and the human–computer interaction science, and is experienced in research into the development and evaluation of Internet-administered support: the group is unique and well equipped to further our knowledge of important aspects of clinically relevant and usable Internet-administered support interventions. We have developed a complex intervention [23] consisting of Internet-administered support (“Carer eSupport”) for ICs of patients with HNC [24]. The first steps were to strengthen the relevance and usability of Carer eSupport, to test the feasibility of Carer eSupport, and to test the recruitment procedures (more clearly described in “Methods”). In our initial focus groups with the ICs of patients with HNC, many expressed a need for emotional and practical support from their social network and healthcare [11, 25]. They commonly changed their work and home routines to prioritize caring for the patient, and some neglected their health needs to attend to the needs of the patient. The ICs’ opinions regarding Internet-administered support stressed the importance of trustworthy information, tailored especially to the ICs of patients with HNC, concerning HNC, cancer treatment, caregiving, their well-being, and daily life activities. The possibility of sharing experiences with and learning from peer ICs and healthcare professionals in discussion forums and real-time video meetings was also highlighted as essential to Carer eSupport. Subsequent focus groups with healthcare professionals having expertise in HNC care [25], conducted to facilitate the possible future implementation of Carer eSupport in clinical care, corroborated the ICs’ experiences and perceived needs for support. These professionals believed that current information on the Internet about HNC and its treatment is too general. However, they also emphasized that the experience of symptoms and side effects is individual and may differ substantially between patients. This clarifies that Internet-administered information is a complement to, and not a substitute for, individualized information from healthcare professionals responsible for patient care [26]. Both ICs and healthcare professionals stressed that online discussions among peer ICs need to be monitored by experts to prevent the spread of incorrect information.

This study protocol outlines the methods of investigating the effects of Internet-administered Carer eSupport plus support as usual (SAU) on self-reported preparedness for caregiving, caregiver burden, and well-being in the ICs of patients with head and neck cancer, compared with ICs receiving only SAU.

## Methods

### Design

The RCT constitutes the third step in developing and evaluating this complex intervention, which is based on the United Kingdom Medical Research Council’s complex interventions framework [23]. The design and methods of the two initial steps of the project have been published elsewhere [24]. The project is being conducted in accordance with the Helsinki Declaration [27], has been approved by the Swedish Ethical Review Authority (Dnr 2020–04650/2022-06520-02/2023-02005-02), and has been registered in ClinicalTrials.gov (registration number: NCT06307418). This protocol complies with the Standard Protocol Items: Recommendations for Interventional Trials guidelines on writing protocols [28]. The software packages used for delivering Carer eSupport, collecting data, randomization, and storing research data are installed on encrypted and secured servers at Uppsala University, in accordance with the university’s information security regulations. Personal data will be stored and secured in a separate database, which is compliant with the European Union General Data Protection Regulation (GDPR, 2016/679).

### Hypotheses

The main hypothesis is that ICs receiving Carer eSupport plus SAU (described below) will report greater preparedness for caregiving than will ICs receiving SAU only. Secondary hypotheses are that ICs who receive Carer eSupport plus SAU will report a lower caregiver burden and better self-perceived health, including less anxiety and depression, than will ICs receiving SAU only.

### Sample

One hundred and ten ICs of patients with HNC will be recruited at ear, nose, and throat (ENT), oncology, and radiotherapy clinics at four Swedish university hospitals. The IC will only be approached if the patient gives written consent. The recruitment will run for ten months, starting in summer 2024.

#### Inclusion criteria

The patients with HNC and ICs will be adults (> 18 years). The patients will be about to start or have undergone at most five radiotherapy treatment sessions/fractions. Radiotherapy may be combined with surgery and/or medical oncological treatment. The IC will be identified as such by the patient and may be a spouse/partner, another family member, or a friend.

#### Exclusion criteria

ICs and patients who do not understand and read Swedish or suffer from cognitive impairment will be excluded. In addition, ICs who are deemed to need support or treatment that cannot be provided by Carer eSupport (e.g., psychotherapy or medication) will be excluded and referred to appropriate healthcare.

### Power analysis

The estimated sample size is based on a study by Holm et al. [29], who evaluated the effect of psycho-education on preparedness for caregiving, using the Preparedness for Caregiving Scale as the primary outcome. According to that study, a total sample size of 55 ICs is required to obtain 80% power to detect a medium-size effect on preparedness using multiple regression analysis (f2 = 0.15, alpha = 5%) [29]. However, based on our earlier Internet-administered studies, high attrition (≤ 50%) may be expected [30, 31] due to the ICs’ strained situation and possible unfamiliarity with computers and/or mobile devices. Therefore, we plan to include 110 ICs, i.e., 55 receiving Carer eSupport plus SAU and 55 receiving SAU only.

### Randomization

ICs will be randomized to Carer eSupport plus SAU or SAU only (ratio 1:1). The randomization will be computer generated and done within the Internet-administered data collection tool, after completion of the baseline questionnaires.

### Development and feasibility testing of carer eSupport

The Social Cognitive Theory (SCT) [32] and the Unified Theory of Acceptance and Use of Technology (UTAUT) [33] constitute the theoretical framework for Carer eSupport. Self-efficacy, a core construct of SCT [32], is conceptualized as a person’s self-perceived capacity to perform in a particular situation. For the ICs of patients with HNC, it may be assumed that ICs with a high self-efficacy regarding their capacity to care for the patient also perceive high preparedness for caregiving, and therefore will be more successful in caregiving. The SCT also states that individuals learn from social interactions with others, which is made possible in Carer eSupport discussion forums and real-time video meetings (described below). The UTAUT is a technology acceptance theory comprising the key constructs of performance expectancy, effort expectancy, social influence, and facilitating conditions. In the context of Carer eSupport, these constructs refer to whether ICs believe that Carer eSupport can improve their preparedness for caregiving, the efforts they expect when using Carer eSupport, whether essential persons in their surroundings believe that they should use Carer eSupport, and organizational or technical enablers of and barriers to using Carer eSupport.

We used the theoretical framework, the results of focus group discussions (presented in “Background”), and existing scientific evidence to develop the first version of Carer eSupport in collaboration with healthcare professionals and an expert group including the ICs of patients with HNC. Thirty-two ICs of patients with HNC have tested that first version in a feasibility study conducted in 2023. The ICs had access to Carer eSupport for one month, after which they completed the questionnaires that will be used in the RCT (described below). Nineteen of 32 ICs took part in individual interviews after 1–2 months regarding their experience of Carer eSupport and their opinions about how it could be improved. Data from the feasibility study were analyzed according to our predefined feasibility criteria [24], i.e., ≥ 45% gave written consent, ≥ 50% used Carer eSupport, < 30% discontinued participation, ≥ 50% completed the questionnaires, and whether Carer eSupport was considered acceptable, relevant, and usable according to the interviews. The analysis revealed that 32 of 52 approached ICs (62%) gave written consent, 22 of them (69%) used Carer eSupport, according to logged “clicks” on text and video files, 16 (50%) completed the questionnaires, and nine (28%) discontinued participation. The ICs experienced Carer eSupport as relevant, helpful, and usable, according to the interviews. However, they gave suggestions for improving Carer eSupport, for example, adding content and improving website design, which have been implemented accordingly. In addition, important knowledge of the recruitment and data collection procedures was obtained. One important finding was the ICs’ experience that the data collection included too many questions. That led to the decision to exclude a fatigue scale since fatigue is the least significant outcome measure compared with our remaining outcome measures, according to previous research [11, 13]. The conclusion was that Carer eSupport and the associated research procedures are feasible after the improvements and can be employed in the RCT. The feasibility study will be reported in detail elsewhere.

### Carer eSupport evaluated in the RCT

Carer eSupport is additional to SAU (described below) and will last for 18 weeks for each IC randomized to the intervention. They will be informed that their access to Carer eSupport will be ended in one week, after 17 weeks via SMS and e-mail. The content of Carer eSupport covers HNC and its treatment, patients’ commonly experienced physical and emotional symptoms, side-effects of treatment, everyday caregiving tasks, self-care, and guided self-help for ICs’ emotional distress (Table 1). The functionality includes text in the form of web pages and PDFs, lectures with voice-over, videos, a question-and-answer (Q&A) section including functionality to pose questions to experts, a monitored discussion forum, and functionality for real-time video meetings. Clinical experts (Table 1) will participate in regular video meetings with ICs and answer questions from ICs in the discussion forums and the Q&A section. PDFs, lectures, and videos will be downloadable.

**Table 1:** The content, functionality and clinical expertise involvement in Carer eSupport, Internet-administered support directed to informal caregivers (ICs) of patients with head and neck cancer

Content	Functionality	Clinical expertise involvement
Head and Neck Cancer (HNC) Human papillomavirus (HPV)	Text Lecture Discussion Forum Real time video meeting	HNC specialist, physician HNC specialist, nurse
Treatment of HNC - Surgery - Radiotherapy - Chemotherapy - Schedule for post-treatment follow-ups	Text Lecture Film Discussion Forum Real time video meeting	HNC specialist, physician HNC specialist, nurse
Side effects of treatment - Nutrition - Oral hygiene - Voice and Speech - Pain - Fatigue - Physiotherapy - Sexuality - Skin - Lymphedema	Text Lecture Discussion Forum Real time video meeting	Dietician Dental hygienist Speech therapist Pain Medicine Specialists HNC specialist, physician HNC specialist, Nurse Physiotherapist Sexologist
Medical devices - Peripherally Inserted Central Catheter (PICC) line, Subcutaneous Venous Port, and Peripheral Venous Catheter (PVC) - Nasogastric tube, Percutaneous endoscopic gastrostomy (PEG), and Radiologically inserted gastrostomy (RIG)	Text Lecture Discussion Forum Real time video meeting	HNC specialist, physician HNC specialist, nurse Dietician
Emotional reactions patients - Crisis - Anxiety - Depression	Text Lecture Discussion Forum Real time video meeting	Healthcare counsellor Psychologist
Emotional reactions ICs - Crisis - Anxiety - Depression - Sexuality	Text Lecture Discussion Forum Real time video meeting	Psychologist Healthcare counsellor Sexologist
Self-Care for ICs - Fatigue - Sleep - Physical activity - Daily life practical issues	Text Lecture Discussion Forum Real time video meeting	HNC specialist, nurse Physiotherapist Healthcare counsellor
ICs own story	Lecture	ICs

### Support as usual (SAU)

SAU comprises the possibility of ICs contacting a specified nurse with expertise in HNC, at the ENT or oncology clinic, to ask for support. These contacts are commonly restricted to occasional appointments and/or phone calls. Healthcare counselors at the clinics are also available for ICs on request. Also, the Swedish healthcare system is always responsible for recognizing the need to support the children (< 18 years old) of patients with cancer; nurses, physicians, and healthcare counselors commonly coordinate that support within Swedish cancer care. Support groups providing emotional and social support to the ICs of patients with various cancer diagnoses may also be arranged on occasion at the clinics. However, Internet-administered support, tailored to the needs of ICs of patients with HNC, is not available in SAU.

### Procedure

#### Recruitment

**Fig. 1:**
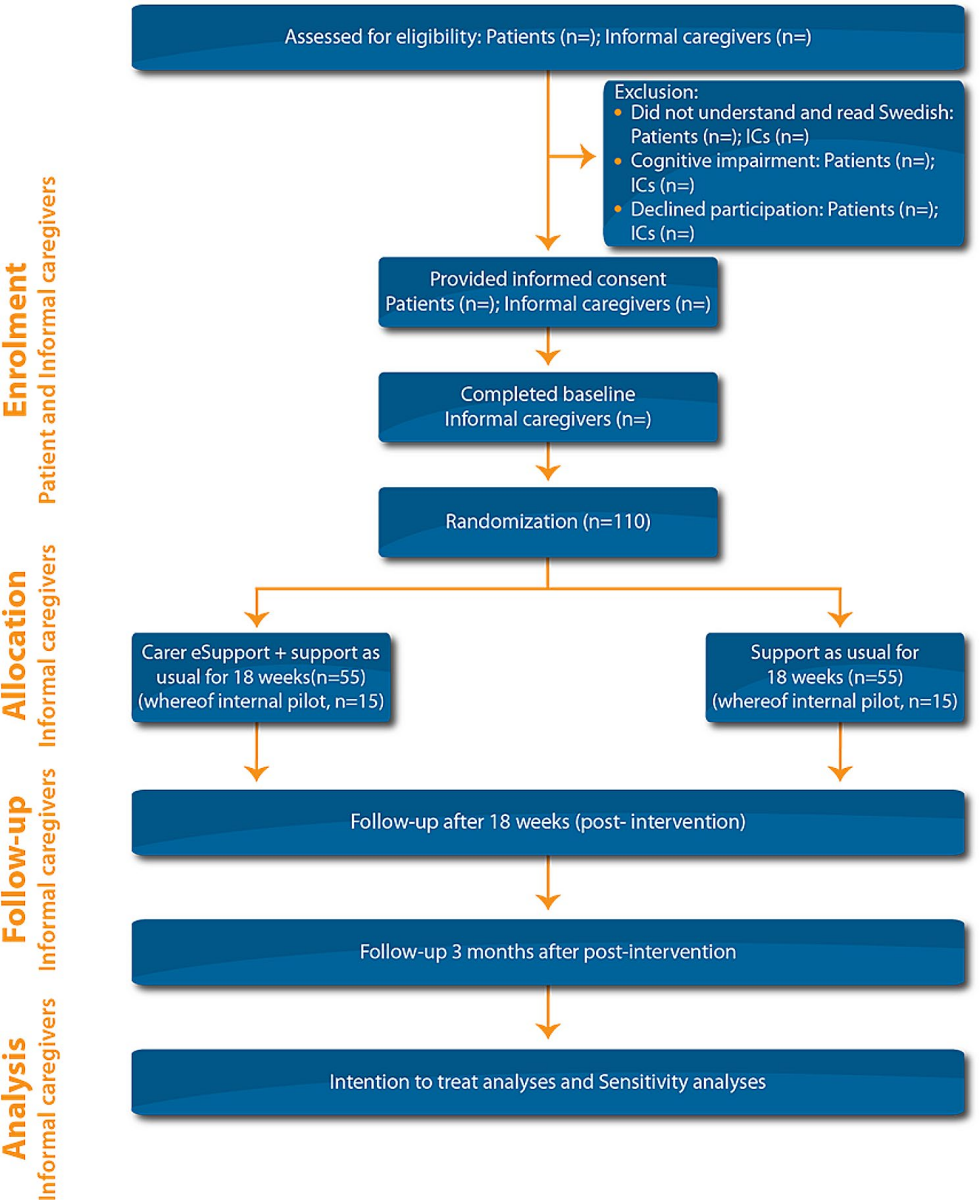
Flow-chart of planned enrollment of patients and informal caregivers (ICs), allocation and follow-up of informal caregivers, and analysis

Nurses at the ENT, oncology, and radiotherapy clinics will identify and screen patients according to the inclusion and exclusion criteria (Fig. 1). A clinical or research nurse will approach an eligible patient in person or by telephone, to inform them about the study, ask for consent to contact an IC (suggested by the patient), and ask whether medical data about the HNC diagnosis and treatment can be collected from the medical record. After the patient has given oral and written informed consent, the clinical or research nurse will contact the IC in person by telephone or email to set a time for the IC to receive information about the study. The information will be given in person, via telephone, or via a video meeting. ICs who give oral and written informed consent will be provided with the web address to the internet-administered questionnaires, a user ID, and a preliminary password. Participating ICs will be randomized to Carer eSupport plus SAU or SAU after the baseline assessment is completed. ICs who are randomized to Carer eSupport plus SAU will be provided with the web address, a user ID, and a preliminary password to Carer eSupport.

#### Allocation and follow-up

Randomized ICs will be allocated to Carer eSupport or SAU for 18 weeks, corresponding to the entire primary treatment period and an additional eight weeks, when the acute side effects are expected to diminish. Follow-up assessments (see “Data collection,” below) are scheduled for 18 weeks after randomization (post-intervention), and three months after post-intervention.

#### Internal pilot study: feasibility testing

ICs included in the internal pilot study will be informed accordingly and asked to provide oral and written consent, using the same procedures as described above. The internal pilot study will comprise a feasibility analysis after the randomization of 30 ICs to confirm the feasibility of the procedures and the updated version of Carer eSupport, completed after the previous feasibility study (described above). The analysis will concern the consent to participate rate, ICs’ use of Carer eSupport according to logged data, and attrition rate one month after randomization. If the progression criteria for feasibility are met [24], i.e., if ≥ 45% give written consent, ≥ 50% use Carer eSupport, and < 30% discontinue participation, the pilot study and the included ICs will seamlessly proceed to the main RCT. However, if the criteria are not met, the recruitment will be ended. ICs randomized to Carer eSupport plus SAU will be able to continue to use Carer eSupport for the entire 18-week period if the recruitment is ended, but they will not be asked to complete the follow-up assessments (see “Data collection”).

### Data collection

Data on primary and secondary outcomes will be collected at baseline, post-intervention (after 18 weeks), and three months after post-intervention. All questionnaire data will be coded and collected using an Internet-administered data collection tool stored and secured on servers at Uppsala University.

Participants will be contacted by telephone by a research nurse if they do not complete the baseline assessment within 1 week, to resolve any potential hindrances to completing the questionnaires; they will subsequently receive reminders via SMS and e-mail if they do not complete the assessment within 1 week, and again after 1 week if it is still not completed. At the assessments post-intervention and three months later, participants will be informed about the assessments one week ahead, via SMS and e-mail, and then be reminded via SMS and e-mail if they do not respond within 1 week after being asked to do so, and again, if still not responding, after 1 more week.

#### Medical and socio–economic background data

Data regarding the patients’ HNC and cancer treatment will be collected from regional healthcare registers, based on clinical data. ICs’ socioeconomic background will be self-reported at baseline, using project-specific questions concerning civil status, parental status, relation to the patient, education level, work situation, economic status, computer skills, and Swedish-language ability.

#### Primary outcome

The Preparedness for Caregiving Scale [34] measures ICs’ perceived preparedness to provide care. It consists of eight items, responded to using 5-point Likert scales ranging from 0 = “not at all prepared” to 4 = “very well prepared.” The items concern the preparedness to: care for the patient, including emergencies (5 items); to find out about and set up services (1 item); to get support from the healthcare system (1 item); and to manage the stress related to being an IC (1 item). The scores are summed to a scale of 0–32, with a higher score reflecting better preparedness. It has been judged to be valid, reliable, and user friendly according to a psychometric evaluation in a Swedish sample of ICs [35].

#### Secondary outcomes

The Caregiver Burden Scale (CBS) [36, 37] comprises 22 items responded to on a 4-point Likert scale ranging from 1 = “not at all” to 4 = “often,” with a higher score indicating more burden. The 22 items can be organized into the following factors, i.e., general strain, disappointment, emotional involvement, environment, and isolation, and the mean of all items indicates the total burden. The CBS is designed to be valid regardless of the patient’s diagnosis and has been used to measure burden among Swedish ICs to patients with various diagnoses.

The RAND-36 [38] is a widely used health-related quality of life questionnaire with good psychometric properties. It measures physical functioning, role limitations due to physical and emotional health problems, social functioning, emotional well-being, energy/fatigue, pain, and general health perceptions. Physical and mental health summary scores may also be calculated. Current Swedish reference values are available [39].

The Depression Anxiety Stress Scale-21 (DASS-21) [40, 41] comprises three 7-item subscales measuring depression, anxiety, and stress. Each item is scored on a 4-point Likert scale, with higher scores indicating more symptoms. The DASS-21 has good psychometric properties, is deemed feasible for research and clinical practice, and has been used in samples of ICs [42].

#### ICs’ use of Carer eSupport: logged data

The ICs’ activity in Carer eSupport will be logged automatically. Logged data will concern the dates and times of log-ins, duration of logged-in periods, use of (i.e., clicks on) text, lectures, and videos, reading of and participation in discussion forums, and reading of and posed questions in the Q&A section.

#### ICs’ satisfaction with Carer eSupport

A subsample of ICs (*n* = 20) of various genders and ages who have had access to Carer eSupport will be asked to participate in a semi-structured individual interview within 1 month post-intervention. The interview will cover their experiences of the relevance, acceptability, and usability of Carer eSupport. In addition, all ICs randomized to Carer eSupport plus SAU will be asked to complete a project-specific questionnaire at the post-intervention follow-up regarding their satisfaction with and perceived benefits of Carer eSupport.

#### Additional professional support and internet use

ICs randomized to SAU only will be asked whether they have received support from professionals (no, occasionally, regularly) and/or whether they have used the Internet for support during the patient’s disease and treatment, using project-specific questions. ICs randomized to Carer eSupport plus SAU will be asked whether they have received support from professionals and/or via the Internet in addition to Carer eSupport. The questions will be administered post-intervention and 3 months post-intervention.

### Data processing and analysis

The effect of Carer eSupport plus SAU on preparedness for caregiving and secondary outcomes, compared with SAU, will be evaluated by intention to treat (ITT) analyses. Linear regression models, mixed-model regression, or ANCOVA will be used. All models will be adjusted for baseline measures, gender, and age. The multiple imputation technique will be used to impute the missing data if they can be assumed to be missing at random. Cohen’s *d* will be calculated to measure the effect size. Sensitivity analyses of the impact of noncompliance will include complete cases and per-protocol analyses. Logged data regarding ICs’ use of Carer eSupport will be analyzed using descriptive statistics. According to Braun and Clarke, the individual interviews will be transcribed verbatim and analyzed using thematic analysis [43].

## Discussion

This study protocol outlines an RCT investigating the effects of the Internet-administered Carer eSupport plus SAU on preparedness for caregiving, caregiver burden, and well-being in the ICs of patients with HNC, in comparison with those receiving SAU only. This discussion section synthesizes the protocol’s key points, contextualizes the study’s significance, discusses strengths and potential challenges, and outlines implications for clinical practice and future research.

The significance of this study lies in addressing unmet needs of ICs, who play a crucial role in the care of patients with HNC but often lack adequate support [11, 12]. Lack of preparedness and caregiver burden can harm the ICs and the quality of care they provide to the patients [1, 6, 13]. By evaluating the effectiveness of an Internet-administered support intervention tailored to the specific needs of the ICs of patients with HNC, this study seeks to reduce the gap between research and clinical care.

The strengths of this study include the careful design of Carer eSupport based on focus groups with the ICs of patients with HNC and healthcare professionals with expertise in HNC care [11, 24, 25], and the involvement of healthcare professionals and ICs in the development of the intervention. These features are pinpointed as essential for any possible future implementation of Carer eSupport in routine clinical care but are lacking in existing research regarding support directed to ICs [20]. Our multidisciplinary research group, including expertise in human–computer interaction, is unique and an additional strength, since usability is key to facilitating long-term engagement in Internet-administered support [21, 22]. The results of the feasibility study met the preset criteria for feasibility [24] and identified how Carer eSupport and the study procedures could be improved, thereby increasing the possibility for a productive RCT.

However, several challenges and considerations need to be acknowledged in implementing this study. First, recruiting and retaining participants, particularly ICs who may already be under significant stress, is challenging. High attrition rates are anticipated due to the strained situation of ICs and potential unfamiliarity with digital platforms. The phenomenon of participants who stop using the support or are lost to follow-up is a common challenge in trials evaluating Internet-administered support [44]. Efforts to mitigate these challenges, such as offering user-friendly interfaces and providing adequate support, are crucial. In addition, usage metrics such as logged data and determinants of attrition need to be analyzed, reported, and discussed to further our knowledge regarding this important topic.

If proven effective, Carer eSupport has the potential to significantly improve the well-being of ICs and enhance their preparedness for caregiving. Integrating Internet-administered support interventions into routine clinical care for patients with HNC and their ICs could lead to more comprehensive and holistic care approaches. Findings of this study may contribute to the development of tailored interventions for ICs of patients with other cancer types or chronic illnesses. Future research could explore the long-term effects of Carer eSupport, evaluate its cost-effectiveness, and investigate factors influencing its implementation and sustainability in real-world clinical settings.

In conclusion, this RCT protocol outlines an evaluation of the efficacy of Internet-administered support for the ICs of patients with HNC. By addressing the unmet needs of ICs, this study also has the potential to improve the quality of care provided to patients with HNC and enhance the well-being of not only their ICs but also the patients themselves.

## Data Availability

The researchers will have access to the final trial dataset in the presented RCT. The data will not be openly accessible at the participant level due to the General Data Protection Regulation (2016/679). However, de-identified data may be available for inclusion in systematic reviews and the like, on request.

## Declarations

### Plans for communicating important protocol amendments to relevant parties

Changes in the protocol will be reported to clinicaltrials.gov and to the authority for research ethics that has vetted the study.

### Monitoring

There is no planned interim analysis or external monitoring of this RCT, since as this is a low-risk intervention directed toward informal caregivers (ICs) of patients with cancer, and not toward patients.

### Patient and public involvement

An expert group of ICs each with experience of being an IC to an individual treated for HNC has been involved in developing the intervention and in planning the study’s procedures. The group will also be asked to take part in interpreting the study results. In addition, one member of the research team has experience of being an IC to an individual treated for HNC.

### Dissemination plan

The results will be presented at national and international conferences, in meetings with clinicians, and to patient organizations including families and friends, and will be published in open-access peer-reviewed journals. The Vancouver authorship guidelines will be employed for all publications emanating from the RCT.

## Abbreviations

ANCOVA: Analysis of covariance
Carer eSupport: The intervention
HNC: Head and neck cancer
IC: Informal caregiver
ITT: Intention to treat
RCT: Randomized controlled trial
SAU: Support as usual
SCT: Social cognitive theory
UTAUT: Unified theory of acceptance and use of technology
